# OLFML2A Overexpression Predicts an Unfavorable Prognosis in Patients with AML

**DOI:** 10.1155/2023/6017852

**Published:** 2023-02-22

**Authors:** Xuan Lu, Ying Li, Yan Yang, Wanchuan Zhuang, Xingxing Chai, Chen Gong

**Affiliations:** ^1^School of Life Sciences, Hefei Nomal University, Hefei, Anhui 230031, China; ^2^Fudan University Affiliated Pudong Medical Center, Shanghai 201301, China; ^3^Department of Hematology, Lianyungang Second People's Hospital, Lianyungang, Jiangsu 222000, China; ^4^Department of Geriatric Medicine, Lianyungang Second People's Hospital, Lianyungang, Jiangsu 222000, China

## Abstract

**Background:**

Acute myeloid leukemia (AML) is a malignant clonal disease of the myeloid hematopoietic system. Clinically, standard treatment options include conventional chemotherapy as well as hematopoietic stem cell transplantation. Among them, chemotherapy has a remission rate of 60% to 80% and nearly 50% relapse in consolidation therapy. Some patients have a poor prognosis due to the presence of unfavorable factors such as advanced age, hematologic history, poor prognosis karyotype, severe infection, and organ insufficiency, which cannot tolerate or are not suitable for standard chemotherapy regimens, and scholars have tried to find new treatment strategies to improve this situation. In the pathogenesis and treatment of leukemia, epigenetics has received attention from experts and scholars.

**Objective:**

To investigate the relationship between OLFML2A overexpression and AML patients.

**Methods:**

From The Cancer Genome Atlas, researchers used the data of OLFML2A gene to analyze and study the pan-cancer using R language and then divided the high and low levels of this protein into two groups to study its relationship with the clinical characteristics of the disease. The relationship between the high levels of OLFML2A and various clinical features of the disease was studied with emphasis on the relationship between the high levels of OLFML2A and various clinical features of the disease. A multidimensional Cox regression analysis was also performed to study the factors affecting patient survival. The correlation between OLFML2A expression and immune infiltration through the immune microenvironment was analyzed. The researchers then conducted a series of studies to analyze the data collected in the study. The focus was on the relationship between the high levels of OLFML2A and immune infiltration. Gene ontology analysis was also performed to study the interactions between the different genes associated with this protein.

**Results:**

According to the pan-cancer analysis, OLFML2A was differentially expressed in different tumors. More importantly, the analysis of OLFML2A in the TCGA-AML database revealed that OLFML2A was highly expressed in AML. The researchers found that the high levels of OLFML2A were associated with different clinical features of the disease, and that the expression of the protein was different in different groups. Those patients with the high levels of OLFML2A were found to have substantially longer survival times compared to those with low-protein levels.

**Conclusions:**

The OLFML2A gene is able to act as a molecular indicator involved in the diagnosis, prognosis, and immune process of AML. It improves the molecular biology prognostic system of AML, provides help for the selection of AML treatment options, and provides new ideas for future biologically targeted therapy of AML.

## 1. Introduction

Acute myeloid leukemia (AML) is a malignant disease of the hematological system with strong biological and clinical heterogeneity. Currently, patients are generally stratified according to their cytogenetic and molecular biological findings to predict their treatment outcome [1]. In practice, we have found that the prognosis of patients graded according to the current risk stratification system still has some variability in treatment outcome and survival time for patients in the same risk stratum, suggesting that we should have a more detailed stratification prognosis system. In recent years, studies have found that AML is associated with multiple mutated genes, suggesting that the development of leukemia may be the result of accumulation of mutations in multiple genes [2, 3]. In recent years, with the widespread use of high-throughput sequencing, i.e., second-generation sequencing technology, the role of leukemia-related mutated genes in diagnosis and treatment has been gradually highlighted [4]. Applying sequencing technology, we have successively discovered a variety of mutated genes related to the development and treatment of AML, and a large number of studies of these genes in AML have also emerged.

The OLFML2A (olfactomedin-like 2A) gene is located on chromosome 9q33.3 and encodes a protein also known as “photomedin-1,” which belongs to the OLFM (Olfactomedin, OLFM) family IV [5]. Olfactomedin, an exocrine glycoprotein secreted by the epithelial cells of the olfactory organ and deposited in large quantities on its surface, was discovered in 1991 and was the first member of the OLFM family [6]. More than half of the OLFM family proteins are expressed in the neural tissue [7]. A large body of evidence suggests that OLFM family proteins play an important regulatory role in neurogenesis, neural crest formation, intercellular adhesion, tumor development, and cell cycle regulation [8, 9]. Related studies have shown that the OLFM family proteins are key regulatory molecules of cellular signaling pathways such as the Wnt signaling pathway [10, 11]. There is increasing evidence that the OLFM family proteins play important roles in the normal tissue development and disease development, e.g., myocilin and olfactomedin 2 are key molecules in the development of glaucoma [12], and OLFM4 is associated with the development of common malignancies such as gastric and pancreatic cancers [13, 14].

OLFML2A is a member of OLFM family IV, which contains at least eight exons spanning 37.7 kb and encodes a protein with an olfactomedin structural domain at the C-terminus and a unique serine/threonine region that distinguishes it from other proteins in the family, two to three potential glycosylation sites at the N-terminus, and homodimers or oligomers with disulfide bonds. Northern-blot of different tissue specimens from mice showed that OLFML2A transcription products were not found in the brain tissue [15]. OLFML2A is an exocrine glycoprotein that binds specifically to chondroitin sulphate-E (CS-E) and heparin [16]. CS-E binds to a number of heparin-binding growth factors, including midkine, Pleiotrophin, several FGFs, and HB-EGF. Specific binding of OLFML2A to CS-E may promote the local action of growth factors bound to CS-E. To date, the specific functions of the OLFML2A gene and its encoded protein remain unknown, and its role in the development of AML has not been reported in the literature.

Based on this, from The Cancer Genome Atlas, researchers analyzed the data using OLFML2A gene data, analyzed and studied pan-cancer using R language, and then divided the high and low levels of the protein into two groups in order to study its relationship with the clinical features of the disease. Finally, it was concluded that the OLFML2A gene, as a molecular indicator, can be involved in the diagnosis, prognosis, and immune process of AML and has the potential to be a reliable prognostic assessment indicator W and a potential therapeutic boot point for AML patients.

## 2. Methods

### 2.1. Preprocessing of Raw Data

We collected TCGA-AML expression profiles and clinical information from TCGA Genomic Data Commons Data Portal (https://portal.gdc.cancer.gov/). We excluded the insufficient cases or missing data in the later information processing. The genomic expression information of OLFML2A was calculated from the TCGA database by high-throughput sequencing. Because all information was publicly available, no ethical approval was required.

### 2.2. Pan-Cancer Analysis

Pan-cancer analysis was performed through the TIMER2 (Tumor Immunology Estimation Resource, version 2) network (https://timer.cistrome.org/) 16 to observe differences in OLFML2A expression in tumors and nearby normal tumor tissue or particular tumor subtypes in the TCGA program.

### 2.3. Gene Expression Analysis

We studied the correlation between different tissue characteristics and OLFML2A expression through the Wilcoxon rank-sum test.

### 2.4. Survival and Clinical Statistical Analysis

The association of OLFML2A with clinical features and overall survival was evaluated using the log-rank tests, Kaplan–Meier survival curves, and one-way Cox analysis. The correlation between high and low OLFML2A expression and clinical features was researched in this study (age, grade, BM blasts, cytogenetic risk, FLT3 mutation, IDH R132 mutation, IDH R140 mutation, NPM1 mutation, PB blasts, RAS mutation, and WBC count) between OLFML2A mutations. In addition, OLFML2A was split into high and low expression groups. The OLFML2A expression was judged in relation to overall survival by confirming the high and low OLFML2A expression based on the median. We applied survROC software to measure the accuracy of risk scores on prognosis using time-dependent subject operating characteristic (ROC) curves. We conducted univariate and multivariate analyses of risk scores after adjusting for age, sex, race, BM blasts, PB blasts, Cytogenetic risk, and FAB classifications. In addition, we analyzed the expression of OLFML2A with different clinicopathological features, and we investigated the association of OLFML2A expression with BM cells, cytogenetic risk, FAB classification, IDH1 R132 mutation, IDH1 R132 mutation, NPM1 mutation, and race.

### 2.5. Construction of Nomograms

Since the development of nomograms, they have been used to forecast cancer prognosis. This method uses a statistical method to score various factors, such as age, gender, and the TNM stage. It can then produce a total score that provides a personalized estimate of the likelihood of the disease returning. In the study, the researchers used nomograms to predict the likelihood of patients with cancer returning. The R package rms generated them. The nomograms were then validated by implementing a series of calibration curves. Subsequently, we utilized the *c*-index to estimate nomogram accuracy.

### 2.6. Immunological Analysis

The researchers then performed a series of studies to analyze the data collected from the studies. They first used a computer program known as the CIBERSORT deconvolution to determine the relative composition of immune cells in each sample. Then, they performed an immune differential analysis to study the two groups' immune cell content differences.

### 2.7. Gene Ontology (GO) and Kyoto Encyclopedia of Genes and Genomes (KEGG)

The cluster profile package was used for GO and KEGG enrichment analysis and underlying biological pathways that were likely to adjust the cancer development. They were also able to identify promising signals that could be linked to the disease.

## 3. Results

### 3.1. OLFML2A Had a High Expression in Multiple Cancer Tissues

**Figure 1:**
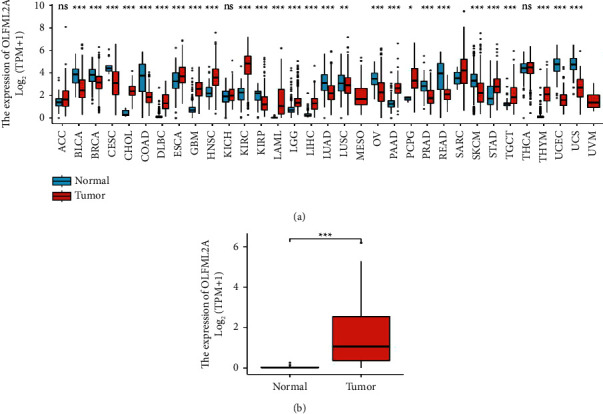
The higher expression of OLFML2A was displayed in AML from the TCGA database.

To detect the differential expression of OLFML2A, we first investigated OLFML2A gene expression in 33 human cancers in TCGA using the TIMER database. Compared with normal samples, OLFML2A had higher expression in 27 cancers, including LUSC, BLCA, DLBC, BRCA, CESC, GBM, COAD, ESCA, KIPR, HNSC, KIRC, LGG, LAML, LUAD, READ, LIHC, PAAD, OV, PRAD, STAD, CHOL, PCPG, SKCM, TGCT, THYM, UCEC, and UCS, shown in Figure 1(a). By comparing the expression of OLFML2A gene in AML and normal samples, OLFML2A had a large overexpression in AML (as shown in Figure 1(b); *P* < 0.001).

### 3.2. The Difference in Clinical Characteristics

**Figure 2:**
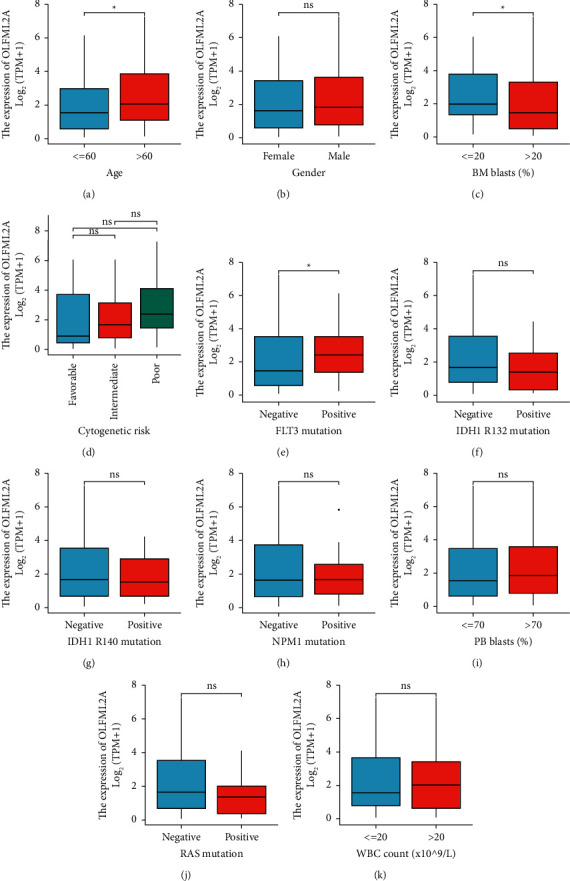
Association between OLFML2A expression and clinical characteristics in the TCGA database.

We grouped high and low OLFML2A gene expression, and the correlation between OLFML2A gene expression and clinical features were explored. We incorporated age, grade, BM blasts, cytogenetic risk, FLT3 mutation, IDH R132 mutation, IDH R140 mutation, NPM1 mutation, PB blasts, RAS mutation, and WBC count for picture mapping. After analyzing the data collected from the studies, the researchers concluded that the high levels of the OLFML2A were different from the low levels of the protein. They also noted that the difference was significant when it came to age, BM blasts, and FLT3 mutations (*P* < 0.05; Figure 2).

### 3.3. The Prognosis and Diagnosis of OLFML2A Value

**Figure 3:**
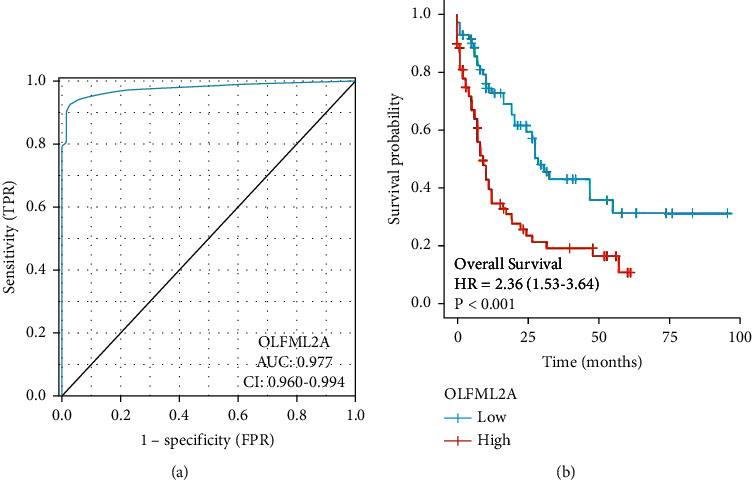
The prognosis and diagnosis of OLFML2A value in TCGA-AML.

Compared with high-risk patients, according to Kaplan–Meier survival curves, the survival of low OLFML2A expression patients was longer (log-rank test; *P* < 0.001) (Figure 3(b)). Figure 3(a) shows 0.977, indicating the area under the curve (AUC) value.

### 3.4. The Difference in Clinicopathological Feature

**Figure 4:**
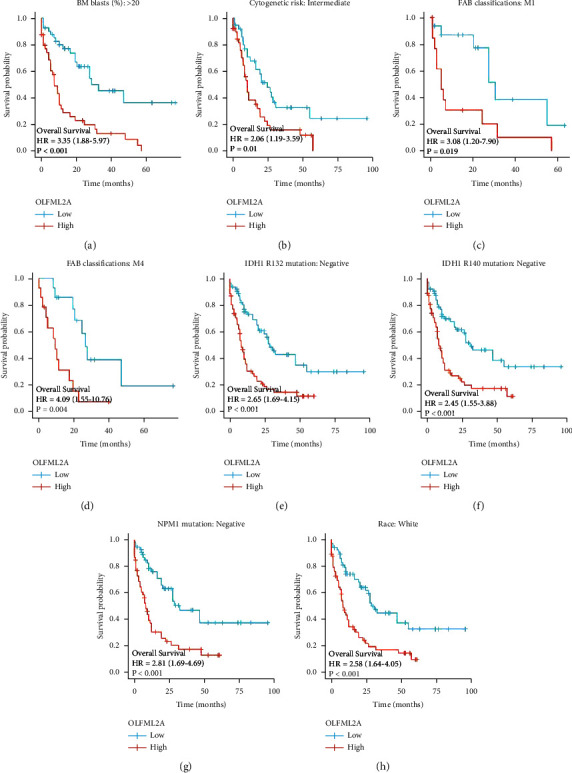
Analysis between OLFML2A expression and different clinicopathological features.

Through the analysis between OLFML2A expression and different clinicopathological characteristics, it could be seen that there was a relationship between OLFML2A expression and FAB classifications, BM blasts, IDH1 R132 mutation, IDH1 R132 mutation, cytogenetic risk, NPM1 mutation, and race (Figure 4 and Tables 1 and 2). In addition, univariate and multifactorial Cox regression analyses presented that an independent risk factor for AML was OLFML2A expression (Table 3).

### 3.5. Construction of the Nomogram

**Figure 5:**
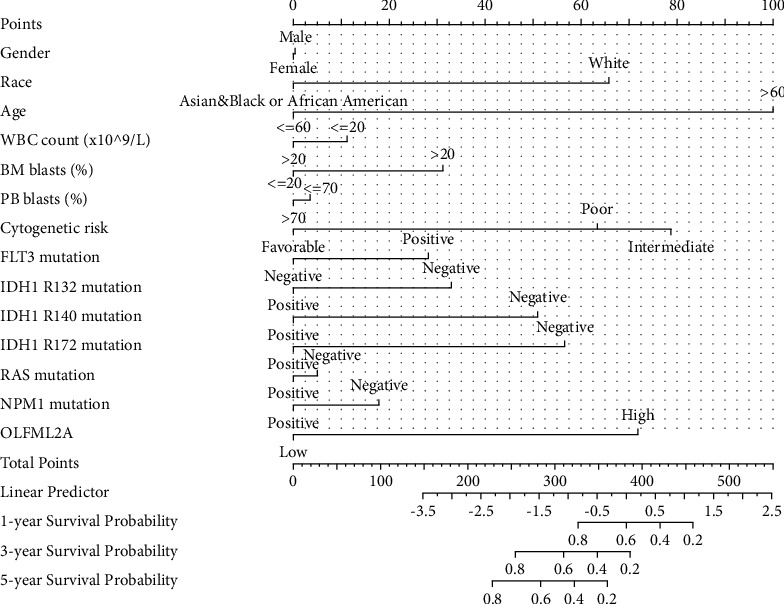
Nomogram for predicting the probability of 1-, 3-, and 5 years OS in TCGA Tumor immune microenvironment.

We constructed a prognostic nomogram in LUAD to predict the 1-, 3-, and 5 year survival probabilities of individuals by gender, race, age, WBC, BM blasts, PB blasts, cytogenetic risk, FLT3 mutation, IDH R132 mutation, IDH R140 mutation, IDH R172 mutation, RAS mutation, NPM1 mutation, PB blasts, and OLFML2A (Figure 5).

**Figure 6:**
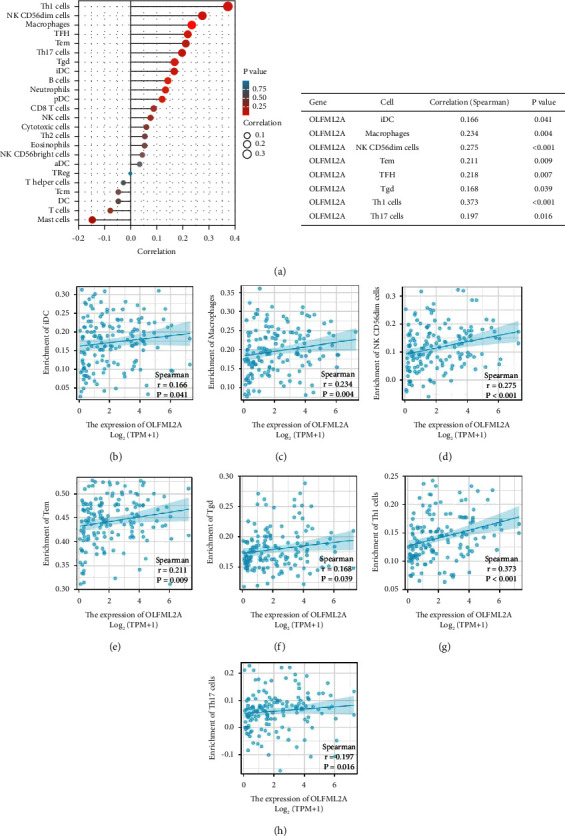
Relationship analysis between OLFML2A expression and immune infiltration in the AML microenvironment.

OLFML2A expression was positively correlated with iDC, macrophages, NK CD56dim cells, Tem, TFH, TGD, TH1 cells, TH17 cells, iDC, macrophages, NK CD56dim cells, Tem, TFH, TGD, TH1 cells, and TH17 cells (*P* < 0.05; Figure 6).

### 3.6. GO and KEGG

**Figure 7:**
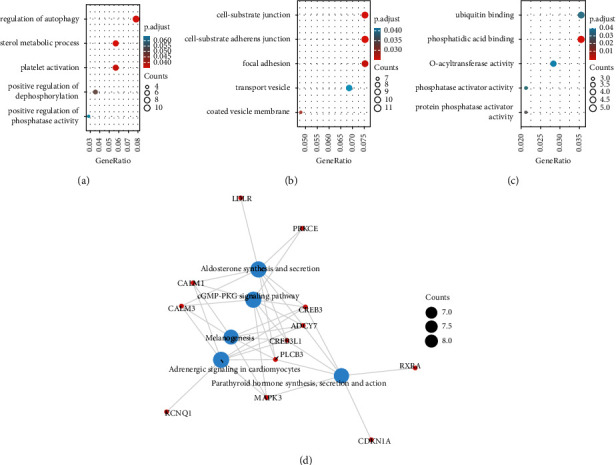
GO and KEGG enrichment analysis of OLFML2A associated genes in the TCGA-AML database.

We performed GO analysis on OLFML2A. CC terms contain “focal adhesion,” “cell-substrate adherence junction,” “cell-substrate junction,” “coated vesicle membrane,” and “transport vesicle”. BP terms include “positive regulation of dephosphorylation,” “regulation of autophagy,” “sterol metabolic process,” “positive regulation of phosphatase activity,” and “platelet activation,”. MF terms were associated with “O-acyltransferase activity,” “ubiquitin binding,” “phosphatase activator activity,” “phosphatidic acid binding,” and “protein phosphatase activator activity”. KEGG analysis shows that OLFML2A is associated with numerous pathways, including “Aldosterone synthesis and secretion,” “cGMP-PKG signaling pathway,” “Melanogenesis,” “Adrenergic signaling in cardiomyocytes,” and “Parathyroid hormone synthesis secretion and action” (Figure 7).

## 4. Discussion

Acute myeloid leukemia (AML) is a common aggressive hematologic malignancy characterized by impaired leukocyte maturation and excessive proliferation of hematopoietic stem cells, which can spread to other organs such as the central nervous system, skin, and gums. Due to impaired normal hematopoietic function, AML patients often present with anemia, bleeding, and severe infections [3]. In the past two decades, genomic, transcriptomic, and epigenomic studies of AML have made great progress. The latest 2017 European Leukemia Network (ELN) risk stratification guidelines combining cytogenetic abnormalities and genetic mutations have been widely used to predict the prognosis of AML patients [4]. Furthermore, based on these advances, several drugs have been approved for the treatment of AML, such as sorafenib for FMS-like tyrosine kinase 3 (FLT3) mutations and Evonib for isocitrate dehydrogenase 1 and 2 (IDH1 and IDH2) mutations [17]. However, most patients with AML who receive chemotherapy relapse [18]. The next step in the approach to treat AML may be to uncover the molecular pathways involved in AML progression, chemotherapeutic efficacy, and relapse, with particular emphasis on the potential role of proteins in AML. There is growing evidence that proteins play an important role in the pathogenesis of cancer, including AML [19].

OLFML2A is an abnormal protein that can be found in various tissues such as the breast, colon, ovary, and liver [1]. Researchers have also found that high levels of this protein are detrimental to patients with acute lymphoblastic leukemia. The researchers hypothesized that the presence of OLFML2A in these patients could help to predict the likelihood of their cancer recurrence. They noted that the high levels of this protein could be a target for novel cancer treatments. In a previous study, researchers found that the presence of OLFML2A in breast cancer cells could hinder the development and metastasis of cancer cells [20]. The knockdown of OLFML2A in glioma cells inhibits the Wnt/*β*-catenin signaling pathway, which leads to upregulation of amyloid precursor protein (APP) expression and a decrease in the degree of stable *β*-catenin, resulting in having reduced MYC, CD44, and CSKN2A2 expression, thereby inhibiting cell proliferation and promoting apoptosis [21, 22].

Furthermore, by exploring the significance of OLFML2A expression in many clinical parameters, we found an association between OLFML2A and AML survival and clinical features. This study performed an immune cell infiltration analysis to gain insight into the role of OLFML2A. From The Cancer Genome Atlas, researchers used OLFML2A gene data to analyze the data. Pan-cancer was analyzed and studied using the R language. They then divided the high and low levels of the protein into two groups to study their relationship with clinical features of the disease. The researchers then conducted a series of studies to analyze the data collected from the study. They focused on the relationship between the high levels of OLFML2A and various clinical features of the disease. They also performed a multidimensional Cox regression analysis to examine the factors that affect patient survival. We analyzed the correlation between OLFML2A expression and immune infiltration in the immune microenvironment. The researchers then conducted a series of studies to analyze the data collected from the study. They focused on the relationship between the high levels of OLFML2A and immune infiltration. They also performed gene ontology analysis to examine the interaction between different genes associated with the protein. OLFML2A was differentially expressed in a variety of tumors based on pan-cancer analysis, including the brain cell counting system, DLBC, ESCA, BRCA, CHOL, LGG, COAD, lipocytes, Kipres, GBM, chronic cell count, oligosaccharide nucleic acid, oligos nucleic acid, adipocyte leukocyte leukemia, adipocyte count enzyme, hyaluronidase, cycloplasmic carcinoma, growth hormone, paclitaxel leukocyte leukemia, prostaglandin, cerebroside leukocyte acid, goitre, and auscocis. In addition, the analysis of OLFML2A in the TCGA-AML database revealed that OLFML2A is highly expressed in AML. The researchers found that high OLFML2A levels were associated with different clinical features of the disease. They also noted that protein expression was different in different groups. Patients with the high levels of OLFML2A were found to survive longer compared to those with low-protein levels. The researchers found that the high levels of OLFML2A were associated with various clinical features of the disease. They also noted that the protein was expressed differently in different groups. Some of these clinical features include BM primitive cells, cytogenetic risk, and IDH1 R132 mutations. Using columnar line graphs, it was possible to predict patient survival based on OLFML2A levels. A relationship was also found between this protein and the growth of acute lymphoblastic leukemia. In the immune microenvironment, the researchers discussed the positive correlation between OLFML2A and various immune cell activities. In parallel, we completed a GO analysis. The CC terminology encompasses “encapsulated vesicle membrane,” “cell-matrix junction,” “focal adhesion,” “focal adhesion,” and “cell-matrix junction.” BP terms include “autophagy regulation,” “positive regulation of phosphatase activity,” “sterol metabolic process,” and “phosphorylation.”

## 5. Conclusion

The OLFML2A gene is able to act as a molecular indicator involved in the diagnosis, prognosis, and immune process of AML. It improves the molecular biology prognostic system of AML, provides help for the selection of AML treatment options, and provides new ideas for future biologically targeted therapy of AML.

## Figures and Tables

**Table 1:** Association analysis between OLFML2A expression levels and clinicopathologic features in the TCGA-AML database.

Characteristic	Low expression of OLFML2A	High expression of OLFML2A	*P* value
*n*	75	76	
Gender, *n* (%)			1.000
Female	34 (22.5%)	34 (22.5%)	
Male	41 (27.2%)	42 (27.8%)	
Race, *n* (%)			1.000
Asian	0 (0%)	1 (0.7%)	
Black or African American	6 (4%)	7 (4.7%)	
White	67 (45%)	68 (45.6%)	
Age, *n* (%)			0.211
≤60	48 (31.8%)	40 (26.5%)	
>60	27 (17.9%)	36 (23.8%)	
WBC count (×10^9^/L), *n* (%)			0.191
≤20	43 (28.7%)	34 (22.7%)	
>20	32 (21.3%)	41 (27.3%)	
BM blasts (%), *n* (%)			0.444
≤20	27 (17.9%)	33 (21.9%)	
>20	48 (31.8%)	43 (28.5%)	
PB blasts (%), *n* (%)			0.372
≤70	39 (25.8%)	33 (21.9%)	
>70	36 (23.8%)	43 (28.5%)	
Cytogenetic risk, *n* (%)			0.035
Favorable	21 (14.1%)	10 (6.7%)	
Intermediate	40 (26.8%)	42 (28.2%)	
Poor	13 (8.7%)	23 (15.4%)	
FAB classifications, *n* (%)			0.278
M0	6 (4%)	9 (6%)	
M1	17 (11.3%)	18 (12%)	
M2	23 (15.3%)	15 (10%)	
M3	7 (4.7%)	8 (5.3%)	
M4	14 (9.3%)	15 (10%)	
M5	4 (2.7%)	11 (7.3%)	
M6	2 (1.3%)	0 (0%)	
M7	1 (0.7%)	0 (0%)	
Cytogenetics, *n* (%)			0.014
Normal	32 (23.7%)	37 (27.4%)	
+8	7 (5.2%)	1 (0.7%)	
Del (5)	0 (0%)	1 (0.7%)	
Del (7)	3 (2.2%)	3 (2.2%)	
Inv (16)	8 (5.9%)	0 (0%)	
t (15; 17)	5 (3.7%)	6 (4.4%)	
t (8; 21)	4 (3%)	3 (2.2%)	
t (9; 11)	0 (0%)	1 (0.7%)	
Complex	7 (5.2%)	17 (12.6%)	
FLT3 mutation, *n* (%)			0.005
Negative	59 (40.1%)	43 (29.3%)	
Positive	14 (9.5%)	31 (21.1%)	
IDH1 R132 mutation, *n* (%)			0.579
Negative	67 (45%)	69 (46.3%)	
Positive	8 (5.4%)	5 (3.4%)	
IDH1 R172 mutation, *n* (%)			0.245
Negative	72 (48.3%)	75 (50.3%)	
Positive	2 (1.3%)	0 (0%)	
IDH1 R140 mutation, *n* (%)			0.745
Negative	67 (45%)	70 (47%)	
Positive	7 (4.7%)	5 (3.4%)	
RAS mutation, *n* (%)			1.000
Negative	71 (47.3%)	71 (47.3%)	
Positive	4 (2.7%)	4 (2.7%)	
NPM1 mutation, *n* (%)			1.000
Negative	59 (39.3%)	58 (38.7%)	
Positive	16 (10.7%)	17 (11.3%)	

**Table 2:** Logistic analysis of the relationship between OLFML2A expression and the clinicopathological features in the TCGA-AML database

Characteristics	Total (*N*)	Odds ratio (OLFML2A)	*P* value
Gender (male vs. female)	151	1.024 (0.539–1.948)	0.941
Race (White vs. Asian and Black or African American)	149	0.761 (0.239–2.305)	0.630
Age (>60 vs. ≤60)	151	1.600 (0.836–3.090)	0.158
WBC count (×10^9/L) (>20 vs. ≤20)	150	1.620 (0.853–3.106)	0.142
BM blasts (%) (>20 vs. ≤20)	151	0.733 (0.379–1.408)	0.352
PB blasts (%) (>70 vs. ≤70)	151	1.412 (0.745–2.692)	0.292
Cytogenetic risk (intermediate and poor vs. favorable)	149	2.575 (1.140–6.156)	0.027
FAB classifications (M1&M2&M3&M4&M5&M6&M7 vs. M0)	150	0.657 (0.210–1.923)	0.448
Cytogenetics (+8&del (5) &del (7) &inv (16) &t (15; 17) &t (8; 21) &t (9; 11) &complex vs. normal)	135	0.814 (0.413–1.600)	0.551
FLT3 mutation (positive vs. negative)	147	3.038 (1.467–6.541)	0.003
IDH1 R132 mutation (positive vs. Negative)	149	0.607 (0.176–1.912)	0.402
IDH1 R140 mutation (positive vs. negative)	149	0.684 (0.194–2.245)	0.533
RAS mutation (positive vs. negative)	150	1.000 (0.228–4.378)	1.000
NPM1 mutation (positive vs. negative)	150	1.081 (0.498–2.357)	0.844

**Table 3:** Univariate and multivariate Cox regression analysis of factors associated with OS in TCGA-AML.

Characteristics	Total (*N*)	HR (95% CI) univariate analysis	*P* value univariate analysis	HR (95% CI) multivariate analysis	*P* value multivariate analysis
Gender	140				
Female	63	Reference			
Male	77	1.030 (0.674–1.572)	0.892		
Race	138				
Asian and Black or African American	11	Reference			
White	127	1.200 (0.485–2.966)	0.693		
Age	140				
≤60	79	Reference			
>60	61	3.333 (2.164–5.134)	<0.001	2.859 (1.819–4.494)	<0.001
WBC count (×10^9^/L)	139				
≤20	75	Reference			
>20	64	1.161 (0.760–1.772)	0.490		
BM blasts (%)	140				
≤20	59	Reference			
>20	81	1.165 (0.758–1.790)	0.486		
PB blasts (%)	140				
≤70	66	Reference			
>70	74	1.230 (0.806–1.878)	0.338		
Cytogenetic risk	138				
Favorable	31	Reference			
Intermediate	76	2.957 (1.498–5.836)	0.002	2.031 (1.003–4.113)	0.049
Poor	31	4.157 (1.944–8.893)	<0.001	2.506 (1.134–5.535)	0.023
FAB classifications	139				
M0	14	Reference			
M1&M2&M3&M4&M5&M6&M7	125	1.033 (0.517–2.062)	0.927		
OLFML2A	140				
Low	71	Reference			
High	69	2.362 (1.534–3.639)	<0.001	2.198 (1.409–3.429)	<0.001

## Data Availability

The experimental data used to support the findings of this study are available from the corresponding author upon request.
